# Multiple Resistance Mechanisms Involved in Glyphosate Resistance in *Eleusine indica*

**DOI:** 10.3390/plants11233199

**Published:** 2022-11-23

**Authors:** Wei Deng, Zhiwen Duan, Yang Li, Cheng Peng, Shuzhong Yuan

**Affiliations:** College of Plant Protection, Yangzhou University, Yangzhou 225009, China

**Keywords:** glyphosate, *Eleusine indica*, target-site resistance, non-target-site resistance, RNA-Seq

## Abstract

Glyphosate is a non-selective herbicide and is widely used for weed control in non-cultivated land in China. One susceptible (S) and five putative glyphosate-resistant (R1, R2, R3, R4, and R5) *Eleusine indica* biotypes were selected to investigate their resistance levels and the potential resistance mechanisms. Based on the dose–response assays, the R3 and R5 biotypes showed a low-level (2.4 to 3.5-fold) glyphosate resistance, and the R1, R2, and R4 biotypes exhibited a moderate- to high-level (8.6 to 19.2-fold) resistance, compared with the S biotype. The analysis of the target-site resistance (TSR) mechanism revealed that the P106A mutation and the heterozygous double T102I + P106S mutation were found in the R3 and R4 biotypes, respectively. In addition, the similar EPSPS gene overexpression was observed in the R1, R2, and R5 biotypes, suggesting that additional non-target-site resistance (NTSR) mechanisms may contribute to glyphosate resistance in R1 and R2 biotypes. Subsequently, an RNA-Seq analysis was performed to identify candidate genes involved in NTSR. In total, ten differentially expressed contigs between untreated S and R1 or R2 plants, and between glyphosate-treated S and R1 or R2 plants, were identified and further verified with RT-qPCR. One ATP-binding cassette (ABC) transporter gene, one aldo-keto reductases (AKRs) gene and one cytochrome P450 monooxygenase (CytP450) gene were up-regulated in R1 or R2 plants. These results indicated that EPSPS overexpression, single or double mutation was a common TSR mechanisms in *E. indica*. Additional NTSR mechanisms could play an essential role in glyphosate resistance. Three genes, ABCC4, AKR4C10, and CYP88, could serve as important candidate genes and deserve further functional studies.

## 1. Introduction

The evolution of herbicide resistance in weeds is an increasingly serious global problem for food security, and herbicide-resistant populations have been documented in up to 263 weed species around the world [1]. Herbicide resistance is a consequence of the weed genetic variability, which can occur in two ways, including either target-site or non-target-site alterations [2,3]. Most cases have been presented for target-site resistance (TSR) to the major groups of herbicides, such as acetolactate synthase inhibitors, acetyl-CoA carboxylase inhibitors, and glyphosate [4,5,6]. Non-target-site resistance (NTSR) can be conferred by any mechanisms that minimize the quantity of herbicide reaching the target-site protein [7]. NTSR involves in various multi-gene families, including cytochrome P450 monooxygenases (P450s), glutathione S-transferase (GSTs), ATP-binding cassette (ABC) transporters, aldo-keto reductases (AKRs), and multidrug and toxic compound extrusion (MATE) [8,9]. For NTSR, it is challenging to determine the molecular basis, and it generally provides low to modest levels of herbicide resistance. However, relatively little attention has been paid to NTSR, especially in a given weed in which TSR is identified.

Glyphosate targets the enzyme of 5-enolpyruvylshikimate-3-phosphate synthase (EPSPS), a key component of the shikimate pathway. Inhibition of the EPSPS enzyme will disrupt the synthesis of aromatic amino acids (tryptophan, tyrosine, and phenylalanine), and result in the subsequent death of the plant [10]. Due to the wide spectrum of control and rapid development of transgenics resistant to glyphosate, this herbicide has become the most widely used herbicide for post-emergence weed control. However, continuous use has resulted in the evolution of glyphosate resistance in at least 53 weed species worldwide [1]. TSR and/or NTSR mechanisms have been discovered in glyphosate-resistant (GR) weeds [11]. The Pro106Ser mutation in the EPSPS was first reported in *Eleusine indica* [12]. Since then, the different mutation types (Pro106His, Pro106Ala, and Pro106Thr) were found in some GR weeds [13,14,15,16]. Subsequently, the Thr102 mutation, the double mutation (Thr102 + Pro106) and even the triple mutation had been reported in GR weeds [17,18,19,20]. In addition, EPSPS gene amplification was also a common TSR mechanism, and had been observed in at least eight GR weed species [11]. NTSR includes reduced glyphosate uptake or translocation, increased glyphosate sequestration to the vacuolar or apoplast, and enhanced glyphosate metabolism, which has been well illustrated in endowing glyphosate resistance [21,22,23,24,25,26]. Moreover, multiple resistance mechanisms may coexist in a single individual or population under the increasing glyphosate selective pressure [26,27,28]. Currently, the underlying molecular mechanisms of glyphosate NTSR has not been fully elucidated, except for in the report by [29,30], which revealed an aldo-keto reductase and an ABCC transporter contribute to the resistance to glyphosate in *Echinochloa colona*. Better understanding the molecular basis of NTSR mechanisms may help to discover novel resistant genes for the breeding of genetically modified crops, while also being useful for formulating a sustainable resistant weed management strategy.

Goosegrass (*Eleusine indica*) is a diploid grass species of the Poaceae family, and it is considered one of the world’s most problematic agricultural weeds. It infests orchards, tea plantations, ridges, and some crop fields, such as cotton, soybean and corn, and glyphosate is widely applied in these cropping systems [27]. However, glyphosate-resistant (GR) goosegrass has been found in Malaysia [31], the Philippines [32], the USA [33], China [34], and Brazil [35]. All resistance mechanisms reported in GR goosegrass are associated with EPSPS single or double mutations, EPSPS overexpression, and point mutations plus overexpression [27,28]. Here, we identified that five goosegrass biotypes (R1, R2, R3, R4, and R5) evolved to be resistant to glyphosate, and both TSR and NTSR mechanisms to glyphosate were investigated.

## 2. Results

### 2.1. Identification of Glyphosate Resistance in Five Resistant Biotypes

**Figure 1:**
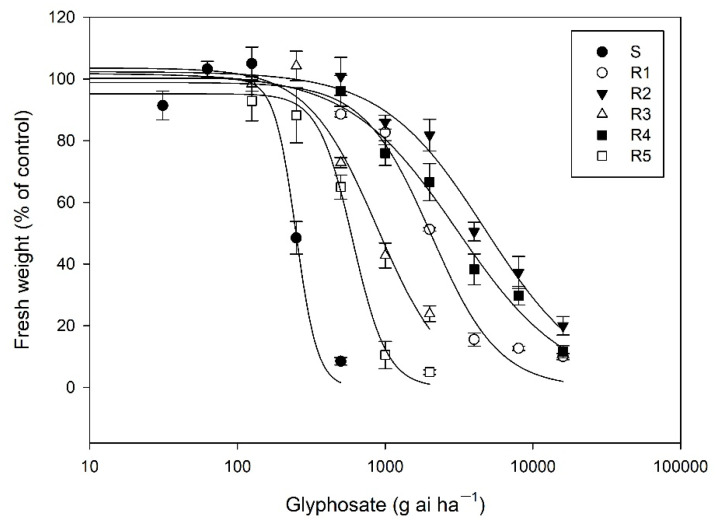
Dose-response curves of six *E. indica* biotypes to glyphosate.

The whole-plant response assays indicated that the five R *E. indica* biotypes showed a varying level (RI = 2.4–19.2) of resistance to glyphosate (Table 1, Figure 1). The S plants was completely killed at rates of 500 g ha^−1^ glyphosate based on our observations, the R3 and R5 plants survived at 1000 g ha^−1^ glyphosate, and the R1, R2, and R4 plants could tolerate a glyphosate rate of 4000 g ha^−1^, respectively, indicating that different glyphosate resistance mechanisms were present in the four R biotypes.

### 2.2. Detection of TSR Mechanisms

To identify the molecular basis of *EPSPS* mutations, a fragment of the *E. indica* EPSPS gene containing the known three resistance-endowing amino acid substitutions (Thr102, Ala103, and Pro106) was analyzed from S and R plants. No amino acid substitutions were found in EPSPS genes of S, R1, R2, and R5 plants. The Pro-106-Ala mutation was observed in the R3 plants, and the heterozygous double mutation (Thr102Ile + Pro106Ile) was detected in the R4 plants (Table 2).

The expression level of *EPSPS* gene was determined in the S and R biotypes. R1, R2 and R5 plants all showed higher level expression (22.4 to 54.1 times) of *EPSPS* gene than the S plants. These results indicated that the Pro106Ala mutation, the double mutation, or overexpression of *EPSPS* gene was the TSR mechanism conferring glyphosate resistance, which was a very common mechanism reported in the R *E. indica* [27,28].

### 2.3. RNA-Seq and De Novo Assembly

In order to obtain comprehensive transcripts of goosegrass, a pooled cDNA library of eighteen mixed samples of RNA from goosegrass seedlings was analyzed on a Majorbio Illumina platform. The libraries generated 980, 134, 648 raw reads (Table S1). We obtained 971,914,934 clean reads after the data clean-up. More than 98.15% of all the raw reads used for the de novo assembly had Phred-like quality scores at the Q20 level (an error probability of 0.02%). We obtained 45,289 transcripts (>200 bp) with an N50 of 2318 bp, and the average length of 1406.78 bp, 79,118 unigenes (>200 bp) with an N50 of 2454 bp and an average length of 1683.3 bp were obtained. The data for the length distribution of the unigenes and transcripts showed the unigine number reduces as the length of the unigenes increases (Table S2).

### 2.4. Sequence Annotation and Classification

**Figure 2:**
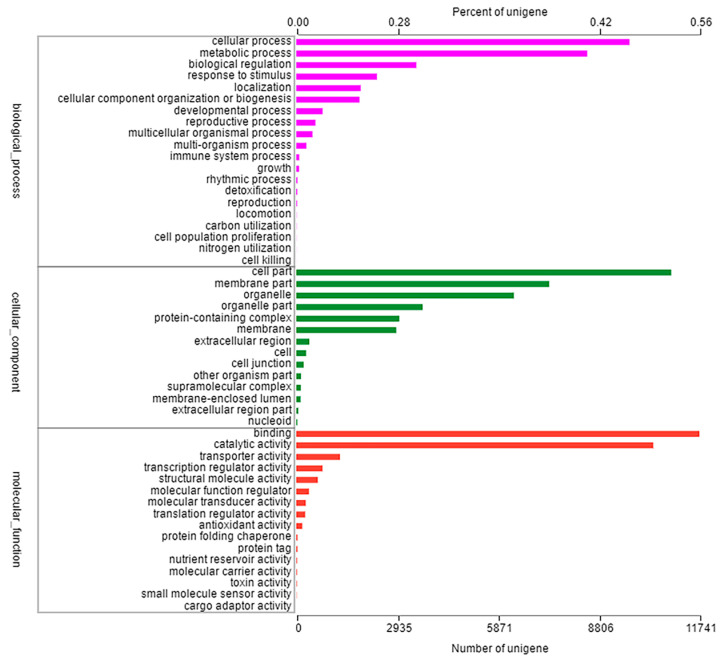
Classification of GO function annotation in *Eleusine indica*. The results are summarized in three main categories: cellular component, molecular function, biological process.

All unigenes obtained by BLAST searches were compared with six major databases (NR, Swiss-prot, Pfam, COG, GO and KEGG databases), we obtained all functional annotation of the transcriptome. In total, 91,743 unigenes were classified into 50 functional categories to at least one GO term, which were divided into three main functional categories: cellular component, molecular function, biological process. The largest proportion of the annotated genes in the cellular component was attributed to cellular process (9704 unigenes, 46.29%) and metabolic process (8474 unigenes, 40.43%); in the molecular function category, the largest proportion of the annotated genes was cell part (10,920 unigenes, 52.09%) and membrane part (7367 unigenes, 35.18%); in the biological process, binding (11,741 unigenes, 56.02%) and catalytic activity (10,399 unigenes, 49.61%) were the most highly represented (Figure 2). Furthermore, 6875 unigenes were assigned into five categories within the KEGG database, including metabolism (3586 unigenes, 52.16%), genetic information processing (2087 unigenes, 30.35%), environmental information processing (451 unigenes, 0.07%), cellular process (451 unigenes, 0.07%), organismal systems (302 unigenes, 0.04%), and human diseases (26 unigenes, 0.004%) (Figure S1). 

### 2.5. Identification of the Differentially Expressed Genes Involved in NTSR

**Figure 3:**
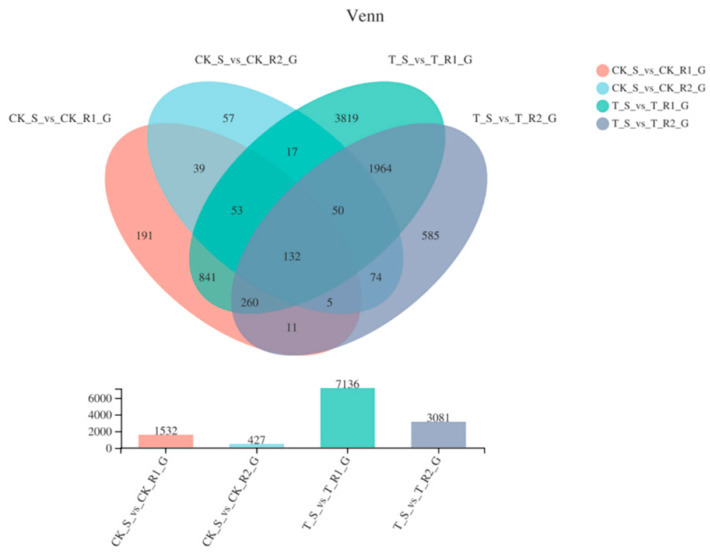
Venn diagram showing the number of DEGs in four samples.

Unigene levels were measured according to RPKM, and the differentially expressed genes (DEGs) for the S, R1, and R2 biotypes were summarized in a venn diagram, which shows their overlapping relationship (Figure 3). A total of 1532 genes were differently expressed between untreated R1 and S samples (p-adjust < 0.05 & |log2FC| ≥ 1), with 731 being upregulated in R1, and 801 being upregulated in S. Besides, 427 genes were expressed in untreated R2 and S samples, where 260 and 167 were upregulated and downregulated in R2 and S, respectively. In total, there were 3071 up-regulated and 4065 down-regulated in treated R1 and S, while there were 1596 up-regulated and 1485 down-regulated in treated R2 relative to S sample (Figure S2). 

Based on the KEGG pathway enrichment and differentially expressed genes analysis, the contigs that were up-regulated between the R1, R2, T-R1, and T-R2 samples, and those that were annotated as NTSR enzymes, were selected as candidate genes. In total, 10 contigs were selected as candidate NTSR genes that may confer glyphosate resistance. Among them, five contigs were annotated to ABC transporter families, two were annotated to AKR families, two were annotated to CytP450 families, and one was annotated to GST families (Table 3).

### 2.6. RT-qPCR Validation of Candidate NTSR-Related Contigs

The expression patterns of the 10 candidate NTSR-related contigs were validated in additional plants from S, R1, and R2 biotypes. Three ABC transporter contigs (DN8045_c0, DN17118_c0_g2, and DN12056_c0_g1), one P450 contig (DN14280_c0_g1), and one GST contig (DN879_c0_g1) showed no difference in expression between CK-S and CK-R1, and CK-S and CK-R2. In addition, two contigs (DN11662_c0_g1 and DN10234_c1) had a variable expression in CK-S vs. CK-R1 and CK-S vs. CK-R2. Importantly, a contig (DN2269_c0) annotated as the ABC transporter ABCC4 was up-regulated in R1, R2, T-R1, and T-R2 samples. The expression of an AKR genes (DN9447_c0) was significantly induced in the R1 and R2 plants after glyphosate treatment. Furthermore, the expression of contigs (DN4583_c0_g2) which were annotated as CYP88, was only induced in the R2 plants. Therefore, only three contigs DN2269_c0 (ABCC4), DN9447_c0 (AKR4C10), and DN4583_c0_g2 (CYP88) were consistently induced or over-expressed in R1 or R2 samples (Table 3).

## 3. Discussion

In this study, the different levels of glyphosate resistance were established in five resistant *E. indica* biotypes. The low or high level of glyphosate resistance was caused by the specific resistance mechanisms. Typically, the R3 biotype with a single Pro106Ala mutation conferred only 3.5-fold resistance to glyphosate, and the R4 possessing the TIPS mutations was 14.2-fold resistant to glyphosate. The high-level resistance attributed to the double mutations has been demonstrated in *E. indica* and other GR weeds [18,36]. In addition, the EPSPS overexpression was found in R1, R2, and R5, while the glyphosate resistance in R1 and R2 was significantly greater than that of R5, suggesting that additional NTSR may be involved. The accumulation of different resistance mechanisms has been a common phenomenon with the intensive and persistent herbicide use [25,26,37]. For example, Alcántara-de la Cruz et al. [26] found that target-site mutations and the reduced translocation endowed the higher glyphosate resistance in *Bidens pilosa* L. Chen et al. [27] reported that the Pro106Ala mutation plus EPSPS overexpression co-evolved in the same plants. These finding indicate that both TSR and NTSR were responsible for glyphosate resistance in R1 and R2. 

RNA-Seq has been conducted to further investigate the NTSR-related genes endowing glyphosate resistance in R1 and R2. A total of 79,118 unigenes and 45,289 transcripts were assembled from 971,914,934 clean reads, and there were 801 and 167 up-regulated contigs in the R1 and R2 samples. Given that the ABC transporters, AKRs, GST, and CYP450 were the well-known important NTSR gene families, a total of 10 contigs were identified from the DEG analysis results. It is noteworthy that some contigs were excluded due to the mismatch between the results of RNA-Seq and RT-qPCR. This difference may be due to the low expression abundance of some genes in plants, or the discrepancy of the two methods. Finally, three contigs, annotated to ABCC4, AKR4C10, and CYP88, were validated by RT-qPCR and selected as candidate NTSR-related genes. 

The ABC transporter families have been implicated in the detoxification of xenobiotics, including herbicides [38,39]. Overexpression of ABC transporter genes (*At*Pgp1 and *ps*NTP9) have been demonstrated to endow resistance to multiple herbicides in *A. thaliana* [40]. In the present study, one contig (DN2269_c0) was annotated to ABCC4 and may be related to glyphosate resistance. In fact, several studies have shown that the ABC transporter genes were involved in glyphosate resistance in GR weeds. Peng et al. [41] reported that two ABC transporters (M10 and M11) play a role in glyphosate NTSR in *Conyza canadensis*. Piasecki et al. [24] identified 19 ABC transporters as NTSR-related candidate genes by transcriptomic analysis in *Conyza bonariensis*. Given the large number of ABC transporter gene families in plants, identification of their function in herbicide resistance has been slow and only Pan et al. [30] revealed that a EcABCC8 gene endows glyphosate resistance in transgenic rice, soybean, and maize. Hence, the candidate ABCC4 genes may play important roles in glyphosate resistance.

AKRs is a multigene superfamily of NAD(P)H-dependent oxidoreductases that mediates detoxification, potassium efflux, and specialized metabolism in plants [42]. There are several examples of AKRs improving glyphosate resistance. Fitzgibbon and Braymer [43] reported that an AKR gene (igrA) from *Pseudomonas* sp. detoxifies glyphosate and confers glyphosate resistance in *E. coli.* Vemanna et al. [44] found that the overexpression of PsAKR1 and OsAKRI in rice and tobacco increased tolerance to glyphosate. Furthermore, the overexpression of EcAKR4-1 can metabolize glyphosate to produce aminomethylphosphonic acid and glyoxylate, which confers a resistance to glyphosate in *Echinochloa colona* [29]. Enhanced glyphosate metabolism has been reported in *Digitaria insularis* and *Conyza canadensis* L. Cronq. [45,46], and it is worthwhile to mine the potential AKR genes mediating glyphosate resistance in these resistant cases. In our study, an AKR gene (AKR4C10) was expressed as glyphosate-induced in both R1 and R2. Moreover, the protein sequence of AKR4C10 from *E. indica* had a 66.1% identity with the reported glyphosate resistance-conferring EcAKR4. Taken together, the AKR4C gene could serve as an important candidate for NTSR genes in *E. indica*.

The role of P450s in herbicide metabolism had been well-known in resistant weeds or tolerant crops. CYP72A31 in rice metabolizes ALS inhibitors bensulfuron-methyl [47]. CYP81A10v7 in *Lolium rigidum* metabolizes herbicides with five modes of action [48]. CYP81A68 in *Echinochloa crus-galli* metabolizes penoxsulam and cyhalofop-butyl [49]. CYP706A3 in *Arabidopsis thaliana* metabolizes dinitroanilines herbicides [50]. However, there is a lack of reports that P450s are involved in glyphosate metabolism or resistance. In this study, a P450 gene (CYP88) was selected as a candidate gene, but the correlation of CYP88 and glyphosate resistance still needs further study.

Herbicide resistance is the consequence of plant evolution and adaption to herbicides. The herbicide selection pressure is an important dynamic for resistance evolution. The TSR and NTSR accumulation in the same biotypes may be due to the higher glyphosate treatment. The normal recommended glyphosate doses have been unable to control the *E. indica*, therefore, the higher herbicide application may result in evolving additional resistance mechanisms. Compared with TSR, NTSR is a greater threat for other herbicides with different sites of action, and this mechanism has been identified in many glyphosate-resistant weed species [25,30,45,46]. Some non-chemical methods, such as physical measures and ecological control techniques, should be encouraged to control the resistant *E. indica* biotypes, rather than the application of chemical herbicides, to avoid the development of multiple resistance.

In conclusion, this study showed that TSR and/or NTSR mechanisms were involved in glyphosate-resistant *E. indica*. The TSR includes the single Pro106Ala mutation, the double TIPS mutation, and EPSPS overexpression, and the NTSR is likely due to ABC transporter (ABCC4)-mediated sequestering glyphosate into apoplasts and AKR (AKR4C10) or P450 (CYP88)-mediated glyphosate metabolism. It is necessary to use transgenic plants to characterize the function of candidate genes. This study needs a further understanding of the glyphosate resistance mechanisms, and a search for relevant NTSR-related genes. 

## 4. Materials and Methods

### 4.1. Plant Materials and Whole-Plant Dose-Response to Glyphosate

The five putative resistant (R) *E. indica* biotypes (Table 1) were collected from the different ridges of paddy fields in Yangzhou city, China. In recent years, local farmers responded that glyphosate had a bad control effect on *E. indica* in this areas, and other non-selective herbicides such as glufosinate-ammonium and diquat were a good choice for the control of *E. indica*. The susceptible (S) biotype was collected from wastelands in Yangzhou city, where no known herbicides were used.

Seeds of *E. indica* were germinated in petri dishes with moistened filter paper for 2 to 3 d, and germinating seedlings were then transferred into 7 cm diameter plastic pots (12–16 plants per pot) filled with fine soil and cultivated in an artificial chamber (30 °C/25 °C, 12: 12 h light/night). When plants had grown to the five- to six-leaf stage, they were thinned to 9 plants per pot and sprayed with different rates of glyphosate (41% glyphosate isopropylamine salt; Roundup, Shanghai, China) using a cabinet sprayer delivering a volume of 450 L ha^−1^ at 200 kPa. The glyphosate rates were 0, 31.25, 62.5, 125, 250, and 500 g ai ha^−1^ for the S biotype, 0, 125, 250, 500, 1000, and 2000 g ai ha^−1^ for the R3 and R5 biotypes, and 0, 500, 1000, 2000, 4000, 8000, and 16,000 g ai ha^−1^ for the R1, R2, and R4 biotypes. The above-ground fresh weight was recorded 2 weeks after treatment. Each treatment contained three replicate pots, and the experiment was independently repeated twice. Because two repeated experiments showed no significant difference (*p* > 0.05), data from two experiments were pooled for calculating 50% growth reduction (GR_50_) using the following logistic regression model with the Sigma Plot 12.2 software:

*y* = C + (D − C)/[1 + (*x*/GR_50_) ^b^

In the model, *y* is the fresh weight response at the glyphosate dose *x*, C represents the lower limit, D represents the upper limit, and b is the slope around GR_50_.

### 4.2. EPSPS Sequencing and Expression

Total DNA was extracted from leaf materials of 10 individuals from each biotype using the Plant Genomic DNA Kit (Tiangen, Beijing, China). For EPSPS sequencing, a pair of primers previously reported by Chen et al. [27] was used to amplify the fragment of *EPSPS* gene containing the known glyphosate resistance-conferring mutation sites. The PCR was performed in a 40 μL reaction system that consisted of 1.5 μL of DNA, 1 μL of each primer (10 μM), 17.5 μL of ddH2O, and 20 μL of Taq PCR MasterMix II (Tiangen). The PCR product was confirmed by 1% agarose gels, and sent to Sangon Biotech company (Shanghai, China) for purification and bi-directional sequencing. The analysis of sequence data was conducted using DNAMAN 5.22 software (Lynnon Biosofe, Quebec, Canada). 

Total RNA was isolated from 6 to 8 plants of each biotype using the MiniBEST Plant RNA Extraction Kit (TaKaRa, Beijing, China) for the detection of EPSPS gene expression. RNA quality was assessed by 1% agarose gels and RNA concentration was measured using a NanoDrop spectrophotometer. An amount of 1 μg of RNA was reverse-transcribed into the first strand of cDNA using the FastKing RT Kit (Tiangen). The β-action gene was used as a reference gene according to a previous report, and the primers were: 5′-AACAGGGAGAAGATGACCCAGA-3′ (forward) and 5′-GCCCACTAGCGTAAAGGGACAG-3′ (reverse). The forward (5′-CTGATGGCTGCTCCTTTAGCTC-3′) and reverse (5′-CCCAGCTATCAGAATGCTCTGC-3′) primers were used for *EPSPS* amplification.

RT-qPCR was performed using the CFX96 Real time PCR System (Bio-Rad) according to the method described in our previous study. The EPSPS expression level relative to the β-action was calculated using the 2^−ΔΔCt^ method.

### 4.3. Sample Preparation for RNA-Seq

The susceptible (S) and glyphosate-resistant (R1 and R2) *E. indica* biotypes were grown to the five-to six-leaf stage, and then treated with glyphosate (1000 g ha^−1^) as described above. Leaf tissues were collected from treated and untreated plants 24 h after treatment, and frozen in liquid nitrogen and stored at −80 °C before RNA extraction. There was no herbicide damage on the leaf tissues 24 h after treatment. Two treatments included untreated control (CK_S, CK_R1 and CK_R2) and glyphosate-treatment (T_S, T_R1 and T_R2), and each treatment had three replicates.

### 4.4. RNA-Seq 

Total RNA extraction and quality control was the same as described above. The cDNA library was constructed, and RNA-Seq was conducted by Meiji Biomedical Technology Co., Ltd. (Shanghai, China). The raw reads were filtered out by quality control to obtain high-quality clean reads. De novo assembly was performed using Trinity. The obtained unigenes were annotated using seven databases (NR, NT, Swiss-Prot, Pfam, COG, GO, and KEGG) with a significance threshold of *E*-value ≤ 10^−5^. The gene expression levels were estimated by fragments per kilobase million mapped reads (FPKM). A differentially expression analysis between different groups (CK_S vs. CK_R1, CK_S vs. CK_R2, T_S vs. T_R1, T_S vs. T_R2, CK_S vs. T_S, CK_R1 vs. T_R1, CK_R2 vs. T_R2) was conducted by DESeq, and genes with an adjusted *p*-value < 0.05 were assigned as differentially expressed genes (DEGs). 

### 4.5. Selection and Validation of Candidate Non-Target Resistance Genes

Candidate unigenes were selected on the basis of up-regulated expression (fold change > 2) in CK_S vs. CK_R or T_S vs. T_R with statistical significance, and annotated to herbicide metabolism genes, especially ABC transporter and AKRs. Plants of untreated S, R1, and R2 and glyphosate-treated S, R1, and R2 were used for RT-qPCR verification of candidate genes. Primers used in qPCR were listed in Table S3 and had an amplification efficiency between 95% and 110%. Each treatment included at least 6 biological replicates. The RT-qPCR was conducted as described above and the data of candidate gene expression levels was analyzed using one-way analysis of variance with Ducan test (*p* < 0.05).

## Figures and Tables

**Table 1:** Geographical location of six *E. indica* biotypes and herbicide rates causing 50% growth reduction (GR50) for the S and R biotypes.

Biotypes	Geographical Location	GR_50_	RI
S	32.36° N, 119.40° E	248.7 ± 13.6	1.0
R1	32.56° N, 119.53° E	2138.6 ± 213.7	8.6
R2	32.33° N, 119.52° E	4763.9 ± 637.7	19.2
R3	32.54° N, 119.22° E	884.2 ± 122.8	3.5
R4	32.63° N, 119.37° E	3531.9 ± 339.7	14.2
R5	32.30° N, 119.41° E	602.9 ± 33.5	2.4

“GR_50_” indicates herbicide rate causing 50% growth reduction of plants. “RI” indicates resistance index, RI = GR_50_(R)/GR_50_(S).

**Table 2:** *EPSPS* genotypes and expression levels in the S and R biotypes.

Biotypes	TSR Mechanisms	
	*EPSPS* Mutations	*EPSPS* Expression
S	No mutations	1
R1	No mutations	22.4 ± 7.3
R2	No mutations	39.0 ± 15.6
R3	Pro-106-Ala	2.5 ± 0.8
R4	Thr-102-Ile + Pro-106-Ile (heterozygotes)	1.7 ± 0.5
R5	No mutations	54.1 ± 6.1

**Table 3:** Selection of up-regulated unigenes annotated and related to glyphosate resistance in *Eleusine indica* biotypes by RNA-Seq and RT-qPCR.

Gene ID	Function Annotation	Fold Change: RNA-Seq				Fold Change: RT-qPCR Validation			
		CK-S/CK-R1	CK-S/CK-R2	T-S/T-R1	T-S/T-R2	CK-S/CK-R1	CK-S/CK-R2	T-S/T-R1	T-S/T-R2
DN8045_c0	ABC transporter, ABCB2	8.3 *	7.3 *	3.1 *	2.0	1.0	0.8	—	—
DN2269_c0	ABC transporter, ABCC4	20.6 *	9.0 *	15.0 *	9.6 *	36.3 *	18.4 *	137.6 *	101.1 *
DN17118_c0_g2	ABC transporter, ABCG50	2.6 *	1.5	1.9	1.1	1.7	0.9	—	—
DN11662_c0_g1	ABC transporter, ABCG11	9.9 *	1.1	1.4	0.7	3.1 *	1.4	—	—
DN12056_c0_g1	ABC transporter, ABCA	6.1 *	2.1	2.3	0.5	0.9	0.4	—	—
DN9447_c0	Aldo-keto reductase, AKR4C10	1.4	1.4	2.8 *	3.7 *	1.7	1.8	3.1 *	3.4 *
DN10234_c1	Aldo-keto reductase, AKR2	3.1 *	1.0	2.4 *	0.6	6.0 *	1.4	—	—
DN4583_c0_g2	CytP450, CYP88	1.5	3.3 *	3.4 *	6.0 *	2.8 *	3.4 *	0.9	3.0 *
DN14280_c0_g1	CytP450, CYP89	6.1 *	2.9	8.9 *	5.5 *	1.3	0.8	0.4	0.9
DN879_c0_g1	GST1	5.1 *	1.2	4.1 *	1.1	0.6	0.7	—	—

“*” analysis shows significant differences (*p* < 0.05), “—” indicates not detected. “CK-S, CK-R1, CK-R2” means untreated control of S, R1, R2 biotypes. “T-S, T-R1, T-R2” means glyphosate treatment of S, R1, R2 biotypes.

## Data Availability

The data are available on request from the corresponding author. The raw Illumina sequence reads have been deposited in the NCBI sequence Read Archive (SRA) database with accession number SRP394458.

## Supplementary Materials

The following supporting information can be downloaded at: https://www.mdpi.com/article/10.3390/plants11233199/s1, Figure S1: Classification of KEGG function annotation Eleusine indica. The *y*-axis lists the various KEGG pathways. The *x*-axis indicates the number of genes. According to participation in KEGG pathways, unigenes were classified into six categories: Metabolism, Genetic Information Processing, Environmental Information Processing, Cellular Process, Organismal Systems and Human Diseases; Figure S2: The number of DEGS between the different groups; Table S1: Quality assessment of sequencing data of *Eleusine indica* (L.) Gaertn.; Table S2: Transcriptional splicing data statistics; Table S3: Primers for fluorescent quantitative of *Eleusine indica* (L.) Gaertn.
