# Supplementary material for: Multiple Resistance Mechanisms Involved in Glyphosate Resistance in Eleusine indica
Source: Plants (Basel). 2022 Nov 23;11(23):3199. doi: 10.3390/plants11233199 (PMC9740094; doi:10.3390/plants11233199)
Supplement: Supplementary file 1 [file plants-11-03199-s001.zip › plants-1944310-supplementary.pdf]

Table S1. Quality assessment of sequencing data of *Eleusine indica* (L.) Gaertn.

Sample	Raw reads	Clean reads	Clean bases	Error rate(%)	Q20(%)	Q30(%)	GC content(%)
CK_R1_1	49273852	48742578	7.26G	0.0245	98.15	94.75	55.51
CK_R1_2	57156216	56682684	8.43G	0.0243	98.26	94.94	56.64
CK_R1_3	55572836	55093634	8.24G	0.0241	98.33	95.12	56.89
CK_R2_1	57667286	57193890	8.51G	0.0242	98.3	95.02	55.95
CK_R2_2	51420504	51009050	7.61G	0.0243	98.25	94.86	56.39
CK_R2_3	55534526	55077946	8.20G	0.024	98.37	95.19	56.65
CK_S_1	53870186	53394964	7.98G	0.0241	98.33	95.15	56.97
CK_S_2	50375378	50002742	7.46G	0.024	98.37	95.21	57.24
CK_S_3	49032528	48661402	7.26G	0.0241	98.36	95.15	55.2
T_R1_1	58414340	57972622	8.64G	0.0241	98.34	95.11	56.43
T_R1_2	56729902	56308918	8.37G	0.0241	98.32	95.07	55.56
T_R1_3	58332378	57808062	8.62G	0.0244	98.22	94.8	55.54
T_R2_1	58248166	57734872	8.60G	0.0244	98.23	94.81	55.59
T_R2_2	55698446	55273330	8.22G	0.0243	98.24	94.85	55.89
T_R2_3	52302004	51757640	7.69G	0.0243	98.25	94.88	54.35
T_S_1	48742902	48312828	7.20G	0.0243	98.26	94.9	55.08
T_S_2	55139630	54707748	8.15G	0.0243	98.28	94.94	54.54
T_S_3	56623568	56180024	8.37G	0.0242	98.32	95.04	55.29

Table S2. Transcriptional splicing data statistics

Assembly quality parameters	Transcripts	Unigenes
200-500bp	12998	14928
500-1kbp	11709	17032
1k-2kbp	9739	21979
＞2kbp	10843	25179
Maximum length	36898	36898
Minimum length	201	201
Average length	1406.78	1683.30
N50 value	2318	2454
N90 value	2978	2410
Total nucleotides	63711510	133179639

Table S3. Primers for fluorescent quantitative of *Eleusine indica* (L.) Gaertn.

Primer	Sequence (5′—3′)
ABCB2-F	GGCGTGGAGTTCCGTTACCC
ABCB2-R	CCCGATCATCATAAGCCTTGCA
ABCC4-F	GGGTTGATGTGGAGGTGAGGA
ABCC4-R	ACAAGCACGTATGACTTCGGAATAT
ABCG50-F	ACTGCTCAGGTTATACTTGCTAC
ABCG50-R	TCGGAAGAATACGGACATTGC
ABCG11-F	GGAGGAGCAGTGGTGAAGAA
ABCG11-R	CGAACCGCAGCCAGTAGTA
ABCA-F	ACAAGCACGTATGACTTCGGAATAT
ABCA-R	GCGACACTTAGCCTTCTCTTC
AKR4C10-F	ACCGCAACGAGAAGGAGGTAGGA
AKR4C10-R	CGGGATGTCGGATGGGATGA
AKR2-F	ATATGCCTAGACTTCGACCAGA
AKR2-R	TCTCAGAATCCCACAAGGTG
CYP88-F	AAACAGGTGCTGATGGATGACGA
CYP88-R	GATGAAGCCGAGGTAGGTGGTG
CYP89-F	AGACGACAACAACGCTGGTGGAG
CYP89-R	CGAACGGCATCATCTTGATCTCC
GST1-F	TGTCTGCTTGGCTAAATGT
GST1-R	CTATCCTCAATCCTGGTCAC

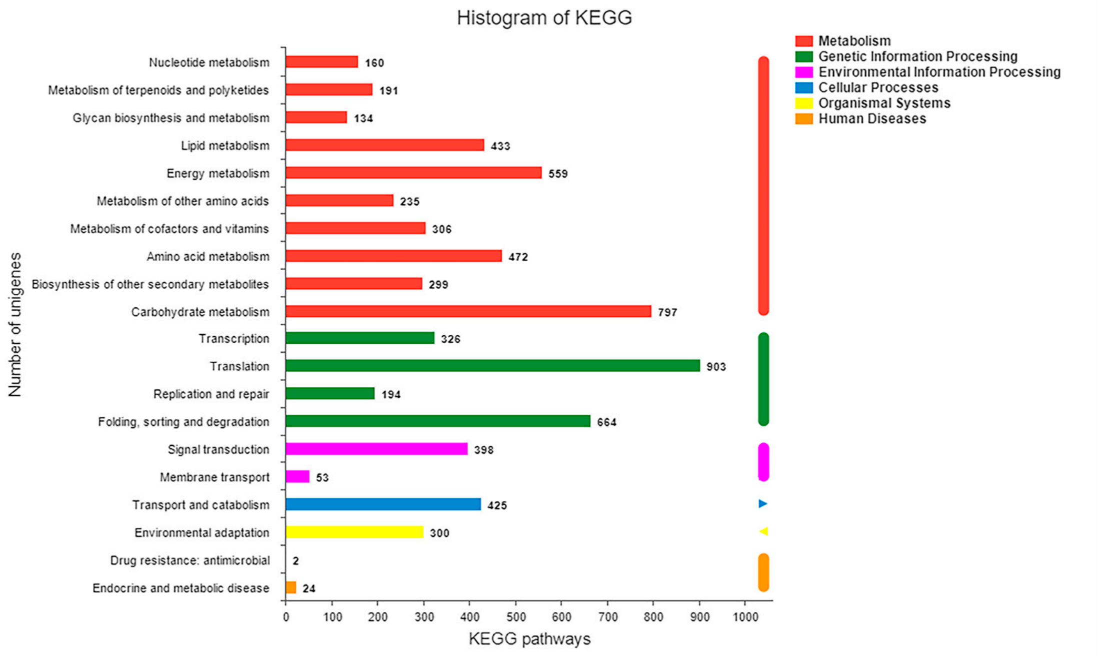

Figure. S1 Classification of KEGG function annotation Eleusine indica.The y-axis lists the various KEGG pathways. The x-axis indicates the number of genes. According to participation in KEGG pathways, unigenes were classified into six categories: Metabolism, Genetic Information Processing, Environmental Information Processing, Cellular Process, Organismal Systems and Human Diseases.

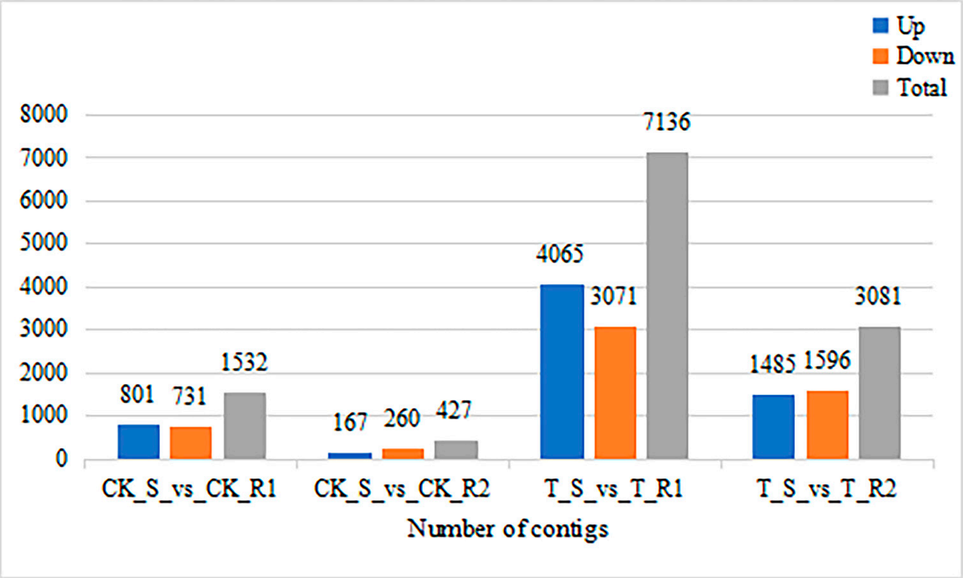

Figure. S2 The number of DEGS between the different groups
